# Adoption of protocols to improve quality of medical research

**DOI:** 10.31744/einstein_journal/2020ED5316

**Published:** 2019-12-10

**Affiliations:** 1 Faculdade de Medicina do ABC, Centro Universitário Saúde ABC, Santo André, SP, Brazil.

The primary means of dissemination and sharing of scientific research results are publications in specialized journals. The quality and relevance of the investigation are assessed, among others, by the material that was published, which is usually the single public register of the research. Problems in this communication hinder the correct evaluation of a study and limit its effectiveness. Such a situation becomes critical becomes critical in a research field such as the health sciences, since it allows wrong decisions to me made by professionals, and consequently, a real potential of harm to the patients.^(^^1^^)^

Medical science requires evidence to identify problems, evaluate the accuracy of the diagnoses or prognoses, compare and assess interventions, describe their adverse or rarest effects, evaluate if an early detection test is really necessary, as well as how to compare intervention costs, among so many other factors. Scientific evidence is produced by means of several experimental approaches and, in general, but not only, by adopting study formats, such as observational studies, randomized studies with intervention, case reports, systematic reviews with meta-analysis, and the opinion of specialists.^(^^2^^)^

**Figure 1:**
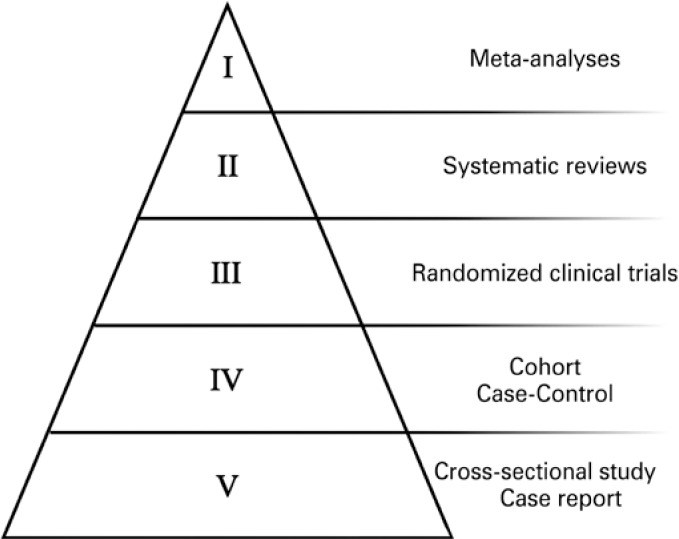
A level of evidence pyramid in medical sciences

The strength, power, or level of evidence in health depends on how this evidence was generated. One form of representation proposed for this evaluation is called pyramid of evidence (Figure 1). This scheme relates research designs with the types of data generated for which the levels of evidence are proposed. At the peak of the pyramid, there are meta-analyses, followed by systematic reviews and randomized clinical trials. The meta-analyses were conceived, initially, as a tool to incorporate robustness into the evidence generated by the randomized clinical trials, for allowing aggregation and comparison of results from independent studies about the estimation of effect sizes of a given intervention. Thus, they enable the clarification of questions derived from independent research, since they expressively widen the sample of investigated subject through data synthesis. Since they are able to provide precise responses, they are considered the studies with the highest level of evidence.^(^^3^^)^

The base of the naturally will have a large amount of data. Typically, there are the shorter, more restrictive studies with limited resources, that have smaller samples or with less restrictive methodological demands than the randomized clinical trials. These are the generically called observational studies, which can be case-control, cohort, ecological, and interventional. They have various origins, but can be, for example, those derived from graduate dissertations and theses. One the other hand, when taken as a whole, these data comprise an expressive volume of subjects, sites, settings, social, economic and genetic characteristics, among others, which are susceptible to pooling.^(^^4^^)^ By means of specific methods, also started the use of observational studies in meta-analyses for decision-making. With this, studies typically from the base of the pyramid, with a low level of evidence and limited impact, began to provide to provide data for the peak of the pyramid, in systematic reviews and meta-analyses.^(^^5^^)^

Nevertheless, one of the greatest challenges for the use of these data is their large heterogeneity, considering the experimental design, report form, selection of research subjects, inclusion and exclusion criteria, and analytical instruments, or the studied populations. In addition to the particularities inherent to smaller and local studies, it is estimated that 85% of data^(^^1^^)^ are wasted due to their low quality, among other reasons.^(^^5^^)^ Therefore, efforts have been made to to increase value to these data and to decrease the information loss, *i.e.,* data that could confer more robustness to the meta-analyses.^(^^6^^)^

In Brazil, scientific production is mostly associated with the research derived from post-graduation programmes. In its majority, there are severe budget constraints, they tend to cover local and regional themes, and therefore, they have low appeal to international - and even to national audiences. This statement is corroborated by the low citation rates of local productions, eloquently exemplified by the fact that 80% of articles published in the field of education in Brazil are not even mentioned by other local projects in the same area.^(^^7^^)^

Whereas in the field of Medicine, Brazil is prolific in terms of absolute volume of scientific production, with 19,636 articles published in 2016, and the 14^th^ country in a productivity ranking in which 130 countries participate. Nevertheless, during the same year, the number of citations per document in the area of medicine was 2.88. Such a fact bestows on Brasil the 107^th^ position in the same ranking in number of citations per article published.^(^^7^^)^

In addition to the low rate of citation, and sometimes, of the poor quality, the Brazilian scientific production still suffers with the great concentration at poles. According to data from The São Paulo Research Foundation (FAPESP - *Fundação de Amparo à Pesquisa do Estado de São Paulo*), more than half the national scientific production comes from the State of São Paulo. In 2017, there were 55,051 publications recorded with researchers of Brazilian institutions, and 42% of them presented with authors from the city of São Paulo. If the State of São Paulo were a country, it would rank 23^rd^ in scientific production.^(^^8^^)^ Normalizing the world mean of citations per publication, which is an index of visibility and impact of publications, São Paulo (1.06) maintains its position above Brazil (0.88),^(^^8^^)^ and still is outstanding by its international collaboration.^(^^9^^)^

Even with its expressive increase in granting master and doctorate titles during the last decade, there was an increase in the production of articles, but there was no improvement of their quality.^(^^10^^)^ This is confirmed by the fact that the number of citations of the Brazilian scientific articles is inferior to that of countries with science-designated budgets lower than those of Brazil.^(^^11^^)^

Nonetheless, there are efforts for improving Brazilian scientific production. Enterprises such as the Brazilian Reproducibility Initiative seek to verify the reproducibility of biomedical studies in various research centers in Brazil.^(^^12^^)^ It is essential that there be a revision of the subject matters and designs of the studies, without disfavoring issues of national interest, but that allow the reproducibility of the study itself, which cooperates to the increase in quality of Brazilian science. Another demand for the refinement of scientific production is the adoption of recommendations of good scientific practices, and increased transparency in the process, in accordance with the growing recommendations recommendations from the international by the international scientific community.^(^^12^^)^

In this scenario, growing prominence is given to research protocols, which are documents aiming to the standardization and, which are the documents that seek standardization and enhancement of the quality of the most diverse types and modalities of scientific research. The use of a protocol can drive data collection, the manner of describing and reporting the data, and even the way in which to structure the investigation from its conception. The protocols recommend which elements and aspects should be observed and considered in research and in its report.^(^^13^^)^ Besides specifying and considering the different types of research, data reveal an increase in the quality of the articles that follow protocols.^(^^14^^)^ Thus, their adoption becomes indispensable for the refinement of national scientific production.

There are several research protocols for different types of studies, with the objective of encompassing demands for the concept of adequate and reproducible experimental study designs. It is important to point out that among the first protocols proposed, some arrived as a demand for studies in the highest levels of the pyramid of evidence. Therefore, taking into account the main types of studies described therein, those that worth mention are the PRISMA,^(^^15^^)^ recommended for the performance of systematic reviews, with or without meta-analysis; CONSORT,^(^^16^^)^ for randomized clinical trials; STROBE,^(^^17^^)^ for cohort and case-control studies; and finally, CARE,^(^^18^^)^ for case report studies. Many others are available, including those encompassing specificities of areas and subareas of research.

Seeking dissemination of information, and as a form of incentive for the use of the protocols, the EQUATOR Network (Enhancing the Quality and Transparency of Health Research) was launched in 2008.^(^^1^^)^ Currently, EQUATOR is headquartered at the University of Oxford, in the United Kingdom, but also has received financial support from organizations, such as the World Health Organization (WHO), Pan American Health Organization (PAHO), National Health Services (NHS), and National Institutes of Health (NIH), being endorsed and recommended by transparency, ethics, and good practices in scientific production and publication agencies, such as the Committee on Publication Ethics (COPE) and the International Committee of Medical Journal Editors (ICMJE), as well as large editorial conglomerates such as BioMed Central, The Lancet, British Medical Journal, PLoS, among others of high and recognized international reputation.^(^^19^^)^ Enhancing the Quality and Transparency of Health Research consists, therefore, of an international collaboration that “aims to improve reliability and value of published literature on health research, promoting transparent and precise reports, and a wider use of robust guidelines in reports.”^(^^20^^)^

In this way, the primary data obtained in observational studies from the base of the pyramid can be better utilized, considered, and therefore, cited when adopting rigorous methodological practices available and in current use. Despite the protocols not guaranteeing the quality of the research per se (*e.g.,* equipment, supplies, and processes), they reinforce the fact that the data were obtained in a premeditated manner, following the best practices adopted by the international scientific community. When their presentation is standardized, relevant information is not omitted, lost, or diluted throughout the text. Thus, they can be better evaluated by reviewers and readers, and will constitute the base of the pyramid of evidence with greater chances of being cited in other studies, especially international ones.^(^^21^^)^ The data may be limited or regional, but it should be of interest and be useful for international science when, for example, such research is selected to compose a synthesis study of scientific literature (systematic review and meta-analysis). For this, it must be strict. Undoubtedly publishing is necessary, but to have an article citation is an acknowledgment of its relevance.

Thus, the adoption of internationally recognized and standardized protocols can foster the strengthening of scientific production and of the dialog among different national and international researchers, increasing the level of citation and acknowledgment of Brazilian science.
