# Supplementary material for: Adoption of protocols to improve quality of medical research
Source: Einstein (Sao Paulo). 2019 Dec 10;18:eED5316. doi: 10.31744/einstein_journal/2020ED5316 (PMC6910067; doi:10.31744/einstein_journal/2020ED5316)
Supplement: Translation

# Translation: Adoção de protocolos para aprimoramento da qualidade da pesquisa médica

**Affiliations:** 1 Faculdade de Medicina do ABC, Centro Universitário Saúde ABC, Santo André, SP, Brasil.

O principal meio de divulgação e compartilhamento dos resultados da pesquisa científica são as publicações em periódicos especializados. A qualidade e a relevância da pesquisa são avaliadas, dentre outros, pelo material que foi publicado, que é quase sempre o único registro público da pesquisa realizada. Defeitos nessa comunicação dificultam a correta avaliação de um estudo e acabam por limitar sua efetividade. Tal situação torna-se crítica na pesquisa em saúde, por permitir decisões equivocadas por profissionais e, consequentemente, com potencial real de danos para os pacientes.^(^^1^^)^

A ciência médica requer evidências para identificar problemas, avaliar a acurácia de diagnósticos ou prognósticos, comparar e avaliar intervenções, descrever seus efeitos adversos ou mais raros, avaliar se um teste de detecção precoce é realmente necessário, bem como comparar custos de intervenções, dentre tantos outros. Evidências científicas são produzidas por meio de abordagens experimentais diversas e, em geral, mas não somente, adotando formatos de estudos, como estudos observacionais, estudos randomizados com intervenção, estudos de casos, revisão sistemática com metanálise e opinião de especialistas.^(^^2^^)^

A força, poder ou nível da evidência em saúde depende da forma como foi gerada esta evidência. Uma forma de representação proposta para esta avaliação é chamada de pirâmide de evidência (Figura 1). Trata-se de um esquema que relaciona os desenhos da pesquisa e os tipos de dados gerados para os quais se propõem níveis de valor de evidência. No topo da pirâmide, encontram-se as metanálises, sseguidas pelas revisões sistemáticas e pelos ensaios clínicos randomizados. As metanálises foram concebidas, inicialmente, como ferramenta para incorporar robustez às evidências geradas por ensaios clínicos randomizados por permitirem a agregação e a comparação de resultados provenientes de estudos independentes sobre a estimativa de tamanho do efeito de uma dada intervenção. Assim, permitem o esclarecimento de questões derivadas de pesquisas independentes, pois ampliam, de forma expressiva, a amostra de sujeitos pesquisados por meio da síntese dos dados. Por serem capazes de fornecer respostas com precisão, são considerados os estudos com o mais alto nível de evidência.^(^^3^^)^

**Figura 1:** A pirâmide de nível de evidência nas ciências médicas

Já na base da pirâmide, naturalmente haverá grande volume de dados. Tipicamente são estudos mais restritos, mais curtos, com recursos limitados, que dispõem de amostras menores, ou com demandas metodológicas menos restritivas que os ensaios clínicos randomizados. São os estudos genericamente chamados de observacionais, que podem ser do tipo caso-controle, coorte, ecológicos e de intervenção. Têm origem diversa, mas podem ser, por exemplo, aqueles derivados de dissertações e teses de pós-graduação. Por outro lado, quando tomados em conjunto, esses dados constituem volume expressivo de sujeitos, locais, settings, características sociais, econômicas, genéticas, dentre outras, que também são passíveis de agregação *(pooling)*.^(^^4^^)^ Por meio de métodos específicos, os estudos observacionais também passaram a ser utilizados em metanálises para tomada de decisão. Com isso, estudos tipicamente da base da pirâmide, com baixo nível de evidência e impacto limitado, também passaram a suprir dados para o topo da pirâmide, em revisões sistemáticas e metanálises.^(^^5^^)^

Entretanto, um dos maiores desafios encontrados para a utilização desses dados é sua grande heterogeneidade, seja de desenho experimental, forma de relato, seleção de sujeitos de pesquisa, critérios de inclusão e exclusão, instrumentos analíticos ou de populações estudadas. Além de particularidades inerentes aos estudos menores e locais, estima-se que 85% dos dados^(^^1^^)^ sejam desperdiçados, dentre outros motivos, por sua baixa qualidade.^(^^5^^)^ Assim, esforços têm sido empreendidos para aumentar o valor desses dados e diminuir o desperdício de informação, que poderia conferir mais robustez às metanálises.^(^^6^^)^

No Brasil, a produção científica está majoritariamente associada à produção derivada de trabalhos de pós-graduação stricto sensu. Em sua maioria, tem severas restrições orçamentárias, tende a abordar temas locais e regionais, e, portanto, apresenta baixo apelo para audiências internacionais – e até mesmo nacionais. Esta afirmação é corroborada pelos baixos índices de citação das produções locais, eloquentemente exemplificada pelo fato de que 80% dos artigos publicados na área de educação no país não são citados nem por outros trabalhos locais da própria área.^(^^7^^)^

Já na área da medicina, o Brasil é produtivo em termos de volume absoluto de publicação científica, contando com 19.636 artigos publicados em 2016, sendo o 14° país em um ranking de produtividade do qual participam 130 países. Entretanto, no mesmo ano, o número de citações por documento na área de medicina foi de 2,88. Tal fato confere ao Brasil a 107^ª^ posição no mesmo ranking em número de citações por artigo publicado.^(^^7^^)^

Além do baixo índice de citação e, por vezes, da baixa qualidade, a produção científica brasileira ainda sofre com a grande concentração em polos. Segundo dados da Fundação de Amparo à Pesquisa do Estado de São Paulo (FAPESP), mais da metade da produção científica nacional é oriunda do Estado de São Paulo. Em 2017, foram registradas 55.051 publicações com pesquisadores de instituições brasileiras, sendo que 42% desse total apresentou autores da cidade de São Paulo. Caso o Estado de São Paulo fosse um país, estaria em 23º lugar no mundo em produção científica.^(^^8^^)^ Normalizando a média mundial de citação por publicação, que é um índice de visibilidade e impacto das publicações, São Paulo (1,06) encontra-se na frente do Brasil (0,88)^(^^8^^)^ e ainda se destaca pela colaboração internacional.^(^^9^^)^

Mesmo com o aumento expressivo na titulação de mestres e doutores na última década, houve aumento na produção de artigos, mas não houve melhoria de sua qualidade.^(^^10^^)^ Este dado é confirmado pelo fato de a quantidade de citações dos artigos científicos brasileiros ser inferior a de países com orçamentos designados à ciência inferiores ao do Brasil.^(^^11^^)^

Entretanto, existem esforços para a melhoria da produção científica brasileira. Empreendimentos como a *Brazilian Reproducibility Initiative* buscam verificar a reprodutibilidade dos estudos biomédicos realizados em diversos centros de pesquisa no Brasil.^(^^12^^)^ É essencial uma revisão das temáticas e dos desenhos dos estudos, sem desfavorecer questões de interesse nacional, mas que possibilitem a reprodutibilidade do estudo em si, o que coopera para o aumento da qualidade da ciência brasileira. Outra demanda para o aprimoramento da produção científica é a adoção de recomendações de boas práticas científicas e de aumento da transparência no processo, em consonância com as crescentes recomendações elaboradas e adotadas pela comunidade científica internacional.^(^^12^^)^

Neste cenário ganham força os protocolos de pesquisa, que são documentos que buscam padronizar e aprimorar a qualidade dos mais diversos tipos e modalidades de pesquisa científica. O uso de um protocolo pode direcionar a coleta de dados, a forma de relatar e reportar os dados e até mesmo estruturar a pesquisa, desde sua concepção. Os protocolos recomendam quais elementos e aspectos devem ser observados e considerados na pesquisa e em seu relato.^(^^13^^)^ Além de especificar e considerar os diferentes tipos de pesquisas, dados mostram aumento na qualidade dos artigos que seguem protocolos.^(^^14^^)^ Assim, sua adoção torna-se imprescindível para o aprimoramento da produção científica nacional.

Existem vários protocolos de pesquisa para diversos tipos de estudos, que visam contemplar as demandas para a concepção de desenhos experimentais adequados e reprodutíveis. Vale ressaltar que, dentre os primeiros protocolos concebidos, alguns surgiram como demanda para os estudos nos níveis mais altos da pirâmide de evidência. Assim, considerando-se os principais tipos de estudos ali descritos, destacam-se o PRISMA,^(^^15^^)^ recomendado para a realização de revisões sistemáticas, com ou sem metanálise; o CONSORT,^(^^16^^)^ para os ensaios clínicos randomizados; o STROBE,^(^^17^^)^ para estudos de coorte e Caso-Controle; e por fim, o CARE,^(^^18^^)^ para estudos de relato de caso. Muitos outros estão disponíveis, inclusive contemplando especificidades de áreas e subáreas de pesquisa.

Visando à propagação da informação, e como forma de incentivo para o uso dos protocolos, foi inaugurado o *Enhancing the Quality and Transparency of Health Research* (EQUATOR Network) no ano de 2008.^(^^1^^)^ Atualmente, o EQUATOR está sediado na *University of Oxford,* no Reino Unido, mas também recebe suporte financeiro de órgãos como Organização Mundial da Saúde (OMS), *Pan American Health Organization* (PAHO), *National Health Services* (NHS) e *National Institutes of Health* (NIH), sendo apoiado e recomendado por órgãos de transparência, ética e boas práticas na produção e publicação científica, como o *Committee on Publication Ethics* (COPE) e o *International Committee of Medical Journal Editors* (ICMJE), bem como grandes conglomerados editoriais, como BioMed Central, The Lancet, British Medical Journal, PLoS, dentre outras instituição de elevada e reconhecida reputação internacional.^(^^19^^)^ O EQUATOR consiste, portanto, em uma colaboração internacional que “procura melhorar a confiabilidade e o valor da literatura de pesquisa em saúde publicada, promovendo relatórios transparentes e precisos e um uso mais amplo de diretrizes robustas de relatórios”.^(^^20^^)^

Dessa forma, os dados primários obtidos em estudos observacionais da base da pirâmide podem ser melhor aproveitados, considerados e, portanto, citados, ao adotar condutas e rigores metodológicos disponíveis e em uso corrente. Apesar dos protocolos não garantirem a qualidade da pesquisa em si (por exemplo: equipamentos, insumos e processo), eles reforçam que os dados foram obtidos de forma planejada, seguindo as melhores práticas adotadas pela comunidade científica internacional. Quando sua apresentação é padronizada, informações relevantes não são omitidas, perdidas e nem ficam diluídas ao longo do texto. Assim, podem ser melhor avaliadas por revisores e leitores, e irão constituir a base da pirâmide de evidência com mais chances de serem citados em outros trabalhos, principalmente os internacionais.^(^^21^^)^ O dado pode ser limitado ou regional, mas ele deve interessar e ser útil para a ciência internacional quando, por exemplo, tal pesquisa é selecionada para compor um estudo de síntese de literatura científica (revisão sistemática e metanálise). E, para isso, deve ser rigoroso. Não resta dúvida de que publicar é necessário, mas ter o trabalho citado é um reconhecimento de sua relevância.

Assim, a adoção de protocolos padronizados e reconhecidos internacionalmente pode propiciar o fortalecimento da produção e do diálogo científico entre diferentes pesquisadores nacionais e internacionais, aumentando o nível de citação e de reconhecimento da ciência brasileira.
